# Phytocytotoxicity of volatile constituents of essential oils from *Sparattanthelium* Mart. species (Hernandiaceae)

**DOI:** 10.1038/s41598-020-69205-6

**Published:** 2020-07-22

**Authors:** Quezia Pains Dutra, Jheniffer Abeldt Christ, Tatiana Tavares Carrijo, Thayllon de Assis Alves, Thammyres de Assis Alves, Luiza Alves Mendes, Milene Miranda Praça-Fontes

**Affiliations:** 1 Postgraduate Program in Genetics and Breeding, Federal University of Espírito Santo, Alto Universitário, s/n, ZIP: 29.500-000, Alegre, ES Brazil; 2 Post Graduate Program in Botany, Department of Botany, National Museum, Federal University of Rio de Janeiro, Quinta da Boa Vista, ZIP: 20.940-040, Rio de Janeiro, RJ Brazil; 3 Department of Biology, Federal University of Espírito Santo, Alto Universitário, s/n, ZIP: 29.500-000, Alegre, ES Brazil; 4 Department of Chemistry and Physics, Federal University of Espírito Santo, Alto Universitário, s/n, ZIP: 29.500-000, Alegre, ES Brazil

**Keywords:** Cell division, Cytogenetics, Environmental sciences

## Abstract

The intensive application of agrochemicals in crops has negatively impacted the environment and other organisms. The use of naturally occurring compounds may be an alternative to mitigate these effects. Plants are secondary metabolite reservoirs and may present allelopathic activity, which is potentially interesting to be used in bioherbicide formulations. In this context, the present work aimed to evaluate the phytotoxic and cytotoxic effects of essential oils extracted from leaves of *Sparattanthelium botocudorum* and *Sparattanthelium tupiniquinorum* in bioassays with the plant models *Lactuca sativa *L. and *Sorghum bicolor *L. Moench. The essential oils were applied at concentrations of 3,000, 1,500, 750, 375 and 187.5 ppm. Chemical characterization of the oils was performed, and their impact on the percentage of germinated seeds, initial development of *L. sativa* and *S. bicolor* seedlings, and changes in the mitotic cycle of meristematic cells from *L. sativa* roots was evaluated. The major compound of the essential oils was germacrene D, followed by bicyclogermacrene, β-elemene and germacrene A. The phytotoxicity assay showed that the essential oils of both species reduced the root and shoot growth in *L. sativa* and decreased the germination and shoot growth in *S. bicolor.* Inhibition was dependent on the tested oil concentration. In the cytotoxicity assay, a decrease in mitotic index and chromosomal and nuclear alterations were observed, which resulted from aneugenic and clastogenic action.

## Introduction

Herbicides, which are used for weed control^1^, are among the most applied agrochemicals in crop fields. However, the use of these compounds has led to problems related to plant resistance, environmental contamination, and risks to human and animal health. Therefore, it is necessary to find alternative methods for agricultural pest control^2,3^. In this context, secondary metabolites may be of particular interest, as they can be directly or indirectly used in the development of new herbicides. These compounds are produced by plants and comprise three major groups, which are found in extracts and essential oils: terpenoids, alkaloids and phenolic compounds^4,5^. Essential oils play an important role in protecting the plant during the competition with other species as well as against herbivore and pest attacks, and are produced variably according to the plant’s interactions with the environment^6^.

Essential oils are volatile products, found in all plant organs, and are obtained by extraction processes such as hydrodistillation and cold pressing, depending on plant location, quantity and characteristics required for the final product^7–9^. The identification and phytochemical classification of these oils enable the investigation of biologically active substances^10–12^. Known as allelochemicals, these substances can be a favorable source for the development of natural herbicides, which may contribute to reduce the environmental impact caused by commercial herbicides, and additionally serve as plant growth stimulants^13–16^.

The basal angiosperm families Hernandiaceae, Lauraceae and Piperaceae are well studied due to presenting essential oils. The species of these families are relevant because they produce secondary metabolites with proven biological activities^17–19^. The genus *Hernandia* (family Hernandiaceae), for example, exhibits important medicinal, anti-inflammatory and blood-purifying properties^20^, as a result of the presence of alkaloids^21,22^.

On the other hand, the genus *Sparattanthelium*, from the same family, remains little known. Only the species *S. amazonum* Mart. and *S. uncigerum* (Meissn) Kubitzki have been characterized. Studies in these species have shown that some of their alkaloids act against resistant and sensitive strains of *Plasmodium falciparum*, a protozoan that causes malaria in humans^23^. In addition, the species described in the literature present medicinal activity, being used by indigenous communities to control digestive problems, stomach pain, vomiting and diarrhea^23,24^.

The evaluation of biological activity and identification of bioactive compounds have been accomplished by means of plant bioassays^4,5,25,26^. Among others, these tests are used to assess phytotoxicity and cytotoxicity as well as to determine the mechanism of action of the studied compounds, and provide results quickly. In addition, model organisms such as *Lactuca sativa* L. (eudicotyledon) and *Sorghum bicolor* L. Moench (monocotyledon) are highly sensitive, enable the simultaneous analysis of various compounds, and exhibit high proliferation rate and rapid germination^5,25,27^.

Considering all the above, this work aimed to (1) determine the chemical composition of the essential oils extracted from leaves of *Sparattanthelium botocudorum* Mart. and *Sparattanthelium tupiniquinorum* Mart., (2) evaluate their phytotoxic properties in bioassay tests, and (3) assess the cytotoxic properties of these oils in *L. sativa*.

## Material and methods

### Plant material

For the extraction of essential oils, *S. botocudorum* and *S. tupiniquinorum* leaves were collected from adult plant, on the same day and time, during winter (July) ,in the south of Espírito Santo, Brazil (21° 07′ 02.5″ S 41°18′ 42.7″ W, data collection: JA Christ) .

For the phytotoxicity and cytotoxicity tests, commercial seeds of the eudicot *L. sativa* (var. crispa) and of the monocot *S. bicolor* (cultivar IAC Santa Elisa) were used.

### Extraction and analysis of essential oils

The extraction method was hydrodistillation in Clevenger apparatus, for 4 h, according to the methodology recommended by Farmacopeia Brasileira for volatile oils^28^. About 200 g of fresh leaves were used in approximately 1 L of reverse osmosis water in a 2-L round-bottom flask. The flask was then coupled to the apparatus, where the oil was extracted for three hours after water boiling. The obtained hydrolate was centrifuged at 5,000 rpm for 3 min to promote the separation between the aqueous and oily phases. With the aid of a Pasteur pipette, the (supernatant) oil was removed and stored in an amber bottle in a freezer at − 20 °C, protected from light^4,29^.

### Phytochemical analysis of the essential oils

Samples of the essential oils extracted from the leaves were analyzed via gas chromatography with flame-ionization detection (GC-FID) (Shimadzu GC-2010 Plus) and gas chromatography–mass spectrometry (GC–MS) (Shimadzu GCMS-QP2010 SE) according to protocol of Souza et al. (2017), with adjustments. For these analyses, the following conditions were used: Helium (He) as carrier gas for both procedures, with flow and linear velocity of 2.80 mL min^−1^ and 50.8 cm s^−1^ for GC-FID and 1.98 mL min^−1^ and 50.9 cm s^−1^ for GC–MS; injector temperature of 220 °C at the split ratio of 1:30; fused silica capillary column (30 m × 0.25 mm) ; stationary phase Rtx^®^-5MS (0.25 μm film thickness) ; oven temperature set as follows: initial temperature of 40 °C for 3 min, then gradual increase at a rate of 3 °C min^−1^ until reaching 180 °C, where it remained for 10 min, for a total analysis time of 59.67 min; and temperatures of 240 °C for FID and 200 °C for MS.

The samples used were removed from the vials in 1 μL volume of a 2% solution of essential oil dissolved in ethanol. The GC–MS analyses were performed on electronic impact equipment with impact energy of 70 eV; scanning interval of 0.50 fragments s^−1^; and fragments detected from 29 to 400 (*m/z*). The GC-FID analyses were performed with a flame formed by H_2_ and atmospheric air at 300 °C, with 40 mL min^−1^ and 400 mL min^−1^ flows for H_2_ and air, respectively. Detection of ions occurs when the organic compounds present in the sample are mixed with the carrier gas (He) and a stream is produced proportional to the amount of these compounds in the sample. If only He and H_2_ are mixed, a small stream is produced between the electrodes.

Identification of the essential oil components was accomplished by comparing the obtained mass spectra with the mass spectra available in spectrographic databases (Wiley 7, NIST 05 and NIST 05 s). In addition, the Kovats Retention Index(KRI) of each compound was calculated using a mixture of C7–C40 saturated alkanes (Supelco-USA) and the adjusted retention time of each compound, obtained via GC-FID, then compared with those in the literature^30–32^.

The relative percentage of each essential oil compound was calculated by the ratio between the total area of the peaks and the total area of all constituents of the sample, obtained by GC-FID analysis. Compounds with a relative area above 1% were identified, and those over 5% were considered to be major^29^.

### Plant bioassays

#### Phytotoxicity assay

For each *S. botocudorum* and *S. tupiniquinorum* essential oil, five concentrations were evaluated: 3,000 ppm, 1,500 ppm, 750 ppm, 375 ppm and 187.5 ppm. The concentrations were obtained by diluting the oil in the solvent dichloromethane (99.5%). Distilled water and dichloromethane were used as negative controls (C−), and glyphosate (1 mL/L) as positive control (C +) . Twenty-five seeds each of *L. sativa* and *S. bicolor* were placed in Petri dishes (9 cm of diameter), with five repetitions, totalling 125 seeds for each treatment. The seeds were placed on filter paper moistened with 2.5 mL of oil diluted in the solvent. Petri dishes were arranged in a completely randomized design (CRD) and placed in Biochemical Oxygen Demand (B.O.D.) at 24 °C throughout the experiment. The percentage of germinated seeds was observed after 8, 16, 24, 36 and 48 h of exposure to the treatments. Root and shoot growth were determined after 48 and 96 h of exposure to the oils, respectively, using a digital caliper. From the data obtained, the following variables were evaluated: germination rate (radicle protrusion) after 48 h (G%) = total of germinated seeds/total of treatment seeds × 100; germination speed index (GSI) = (N1 * 1) + (N2 − N1) * 1/2 + (N3N2) * 1/3 + … (Ny − (Ny − 1)) * 1/y, where *Ny* represents the number of germinated seeds in a given period and *y* represents the total number of evaluation periods^33^; and root length (RL) and shoot length (SL), in mm^25^.

#### Cytotoxicity assay

After 48 h of exposure, the roots of ten lettuce seeds were removed and fixed in Carnoy I (3:1 methanol + acetic acid) , then stored at − 20 °C for at least 24 h. Only lettuce roots were used in this assay, as they are considered a suitable model for microscopic analysis to test the toxic effect of chemical compounds^27^. Lettuce roots show high proliferative activity, fast growth, a high number of seeds, many chromosomes, high sensitivity to mutagenic and genotoxic compounds, and easy-to-manipulate roots^5,34^. Slides were prepared using the squashing method^35^. First, root tips were hydrolyzed in 5 N HCl at room temperature for 18 min. Next, the meristematic region was removed and placed on a slide, stained with 2% acetic orcein, covered with acoverslip, and crushed. Each slide was prepared using two treated meristems, with five slides being evaluated per treatment (one slide for each Petri dish). One thousand cells were evaluated per slide, totaling 5,000 meristematic cells observed per treatment. The following parameters were analyzed: mitotic index (MI) , calculated as the number of dividing cells as a fraction of the total number of cells; chromosomal aberrations (CA) , expressed as the percentage of each aberration—lost chromosomes, adherent chromosomes, chromosomal fragments, chromosomal bridges, c-metaphases and chromosomal polyploidization—divided by the total number of cells; and nuclear aberrations (NC), determined by the frequency of condensed nuclei and micronucleated cells in interphase^25,34^.

### Statistical analyses

The results obtained from the phytotoxicity and cytotoxicity analyses were subjected to analysis of variance, and the means to Dunnett's test at 5% significance. This test was chosen to compare treatments with controls^36^ and because it is sensitive and able to identify small differences between groups^37^. The analyses were performed with Genes, a software package for analysis in experimental statistics and quantitative genetics^38^.

## Results

### Chemical composition of the essential oils

The extraction efficiency of *S. botocudorum* and *S. tupiniquinorum* essential oils was 0.34% and 0.43%, respectively, by fresh leaf fraction. By means of the chromatographic analyses, five compounds were identified in the essential oils of these species (Table 1). In the essential oil from *S. botocudorum*, the major compound was germacrene D (33.2%), followed by bicyclogermacrene (23.4%) , germacrene A (17.7) , β-elemene (8.4%) and trans-nerolidol (7.7%) . The major compound in *S. tupiniquinorum* was also germacrene D (44.8%), followed by bicyclogermacrene (16.9%), γ-cadinene (15%), germacrene A (8.7) and β-elemene (5.1%). Only one chemical compound differentiated the two species, namely trans-nerolidol in *S. botocudorum* and γ-cadinene in *S. tupiniquinorum*.

**Table 1:** Identification of the major compounds (> 5%) of the essential oils from *Sparattanthelium botocudorum* and *Sparattanthelium tupiniquinorum.*

	*S. botocudorum*	*S. tupiniquinorum*
Compounds^a^	A_rel_ (%) ^b^	A_rel_ (%) ^b^
β-Elemene	8.4	5.1
Germacrene D	33.2	44.8
Bicyclogermacrene	23.4	16.9
Germacrene A	17.7	8.7
*trans*-Nerolidol	7.7	–
γ-Cadinene	–	15.0
Total identified	90.4	90.5

^a^Major compounds listed in order of elution using Rtx^®^-5MScolumn.

^b^Compounds with > 5% relative area were identified.

#### Phytotoxicity

**Figure 1:**
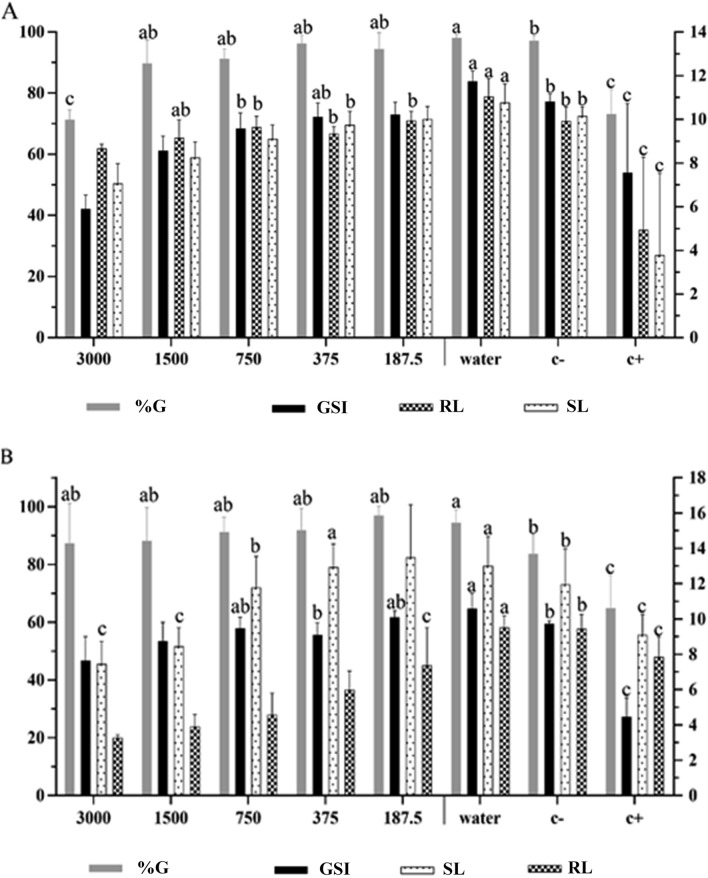
Phytotoxicity of different concentrations of *S. botocudorum* essential oil, water, solvent (C −) and glyphosate (C +) , tested on seeds and seedlings: **(A)** *L. sativa* and **(B)** *S. bicolor*. The means followed by the letter *a* equalled distilled water (C −); means followed by *b* equalled dichloromethane (DCM) (C −); and means followed by *c* equaled glyphosate (C +). The numbers in the figure caption represent the concentrations (ppm) of the essential oils. %G: percentage of germinated seeds after 48 h of exposure to treatments; GSI: germination speed index of the seeds during the first 48 h, evaluated every 8 h, after exposure to treatments; RL: root length (mm) after 48 h of exposure to treatments; SL: shoot length (mm) after 120 h of exposure to treatments. The left y axis refers to the means of germination, and the right y axis to GSI, RL and SL.

**Figure 2:**
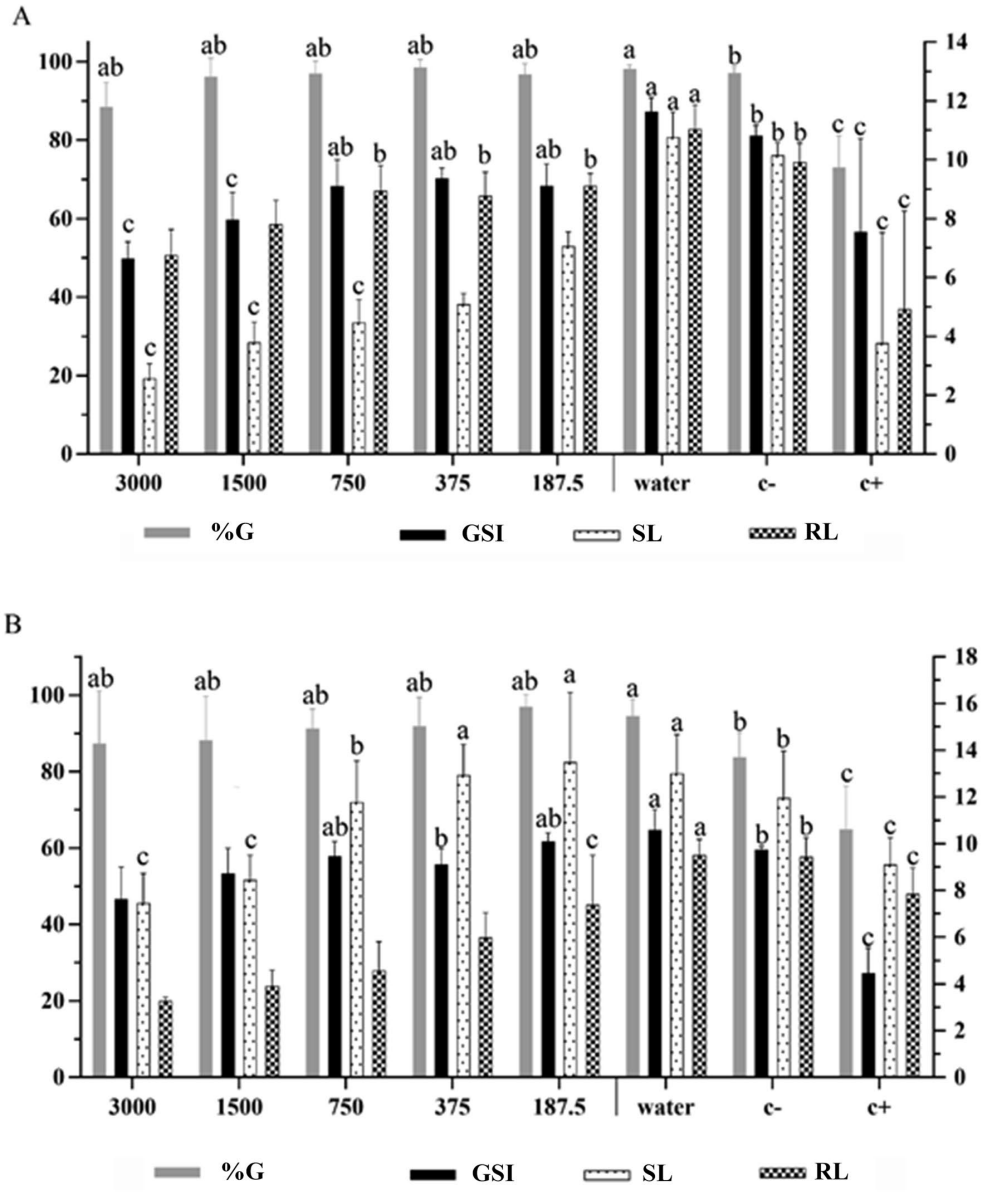
Phytotoxicity of different concentrations of *S. botocudorum* essential oil, water, solvent (C −) and glyphosate (C +) , tested on seeds and seedlings: **(A)** *L. Sativa* and **(B)** *S. bicolor*. The means followed by the letter *a* were equal to distilled water (C −); means followed by *b* were equal to dichloromethane (DCM) (C −); and means followed by *c* were equal to glyphosate (C +) . The numbers in the figure caption represent the concentrations (ppm) of the essential oils. %G: percentage of germinated seeds after 48 h of exposure to treatments; GSI: germination speed index of the seeds during the first 48 h, evaluated every 8 h, after exposure to treatments; RL: root length (mm) after 48 h of exposure to treatments; SL: shoot length (mm) after 120 h of exposure to treatments. The left y axis refers to the means of germination, and the right y axis to GSI, RL and SL.

The essential oils from both *Sparattanthelium* species performed biological activities in the two used plant models. Inhibition was dependent on the analyzed variable and the oil concentration tested (Figs. 1, 2). The essential oil from *S. botocudorum* decreased the germination index of *L. sativa* seeds at the highest concentration (3,000 ppm), matching the positive control (glyphosate) (Fig. 1a). The GSI was also reduced when compared to the positive control, being more expressive at the concentration of 3,000 ppm, and matched the positive control at 1,500 ppm. Root length was not affected, and all germinated seeds produced roots with length similar to those treated with negative controls (water or dichloromethane). On the other hand, at the two highest oil concentrations, shoot length was smaller than the growth observed in the treatments with the negative controls.

For the treatments performed with *S. bicolor* seeds, a germination percentage close to that of seeds exposed to negative controls was observed at all tested concentrations (Fig. 1b). GSI was also unaffected by the essential oils. In turn, root length was the most affected variable, with a reduction more effective than glyphosate observed at all tested concentrations, except at 1,500 ppm. Shoot growth was inhibited at the concentration of 3,000 ppm, presenting means close to the positive control; means for the other concentrations were close to the negative controls.

The essential oil from *S. tupiniquinorum* had no effect on the germination of *L. sativa* seeds (Fig. 2a); treatment means were similar to negative controls. However, GSI was inhibited at the highest concentration, matching the positive control. Root length was also reduced in the seeds exposed to the treatments at 3,000, 1,500, 750 and 375 ppm when compared with the negative controls. For the variable shoot length, it was observed that the treatments with 3,000 and 1,500 ppm of the oil were similar to glyphosate.

In the test with *S. bicolor* seeds, the germination percentage, at all tested concentrations, showed means close to the negative control (Fig. 2b). However, for GSI, the concentration of 3,000 ppm was similar to treatment with glyphosate. Root length was the most affected variable; all concentrations, except 187.5 ppm, inhibited root growth more than glyphosate did. For shoot length, significant inhibition was observed at the two highest concentrations.

#### Cytotoxicity

**Figure 3:**
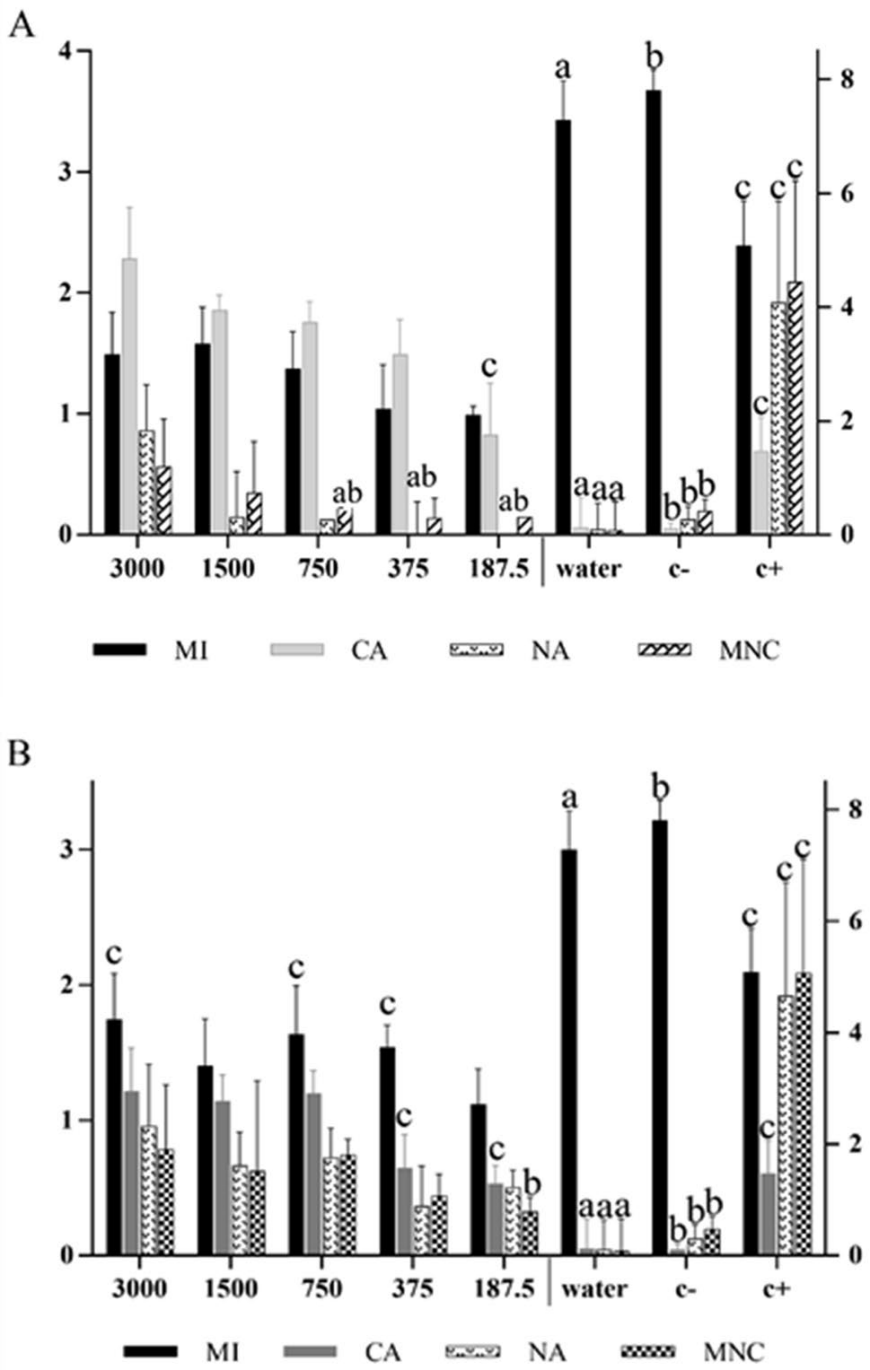
Meristematic cell analysis of *L. sativa* roots treated with different concentrations (ppm) of the essential oils from **(A)** *S. botocudorum* and **(B)** *S. tupiniquinorum*, water (C −), solvent (C −) or glyphosate (C +) . The means followed by the letter *a* equalled distilled water (C −); means followed by *b* equalled dichloromethane (DCM) (C −); and means followed by *c* equaled glyphosate (C +) . The five variables presented are MI: mitotic index; CA: chromosomal alterations; NA: nuclear alterations; and MNC: micronuclei. The numbers in the figure caption represent the concentrations (ppm) of the essential oils. The left y axis shows the values of MNC and MN, and the right y axis refers to the values of CA and MI, according to Dunnett’s test (p < 0.05).

The essential oils from *S. botocudorum* and *S .tupiniquinorum* were cytotoxic to lettuce meristematic cells. A reduction in MI and an increase in CA and NA were observed (Fig. 3a, b). The MI of cells treated with the essential oil from *S. botocudorum* was more affected than by glyphosate. The oil from *S. tupiniquinorum* also acted similarly to glyphosate for MI. As for the chromosomal changes, an increase was observed according to the oil concentration, nearing the positive control at the lowest concentrations and being more toxic at higher concentrations. Nuclear and micronuclear alterations were more expressive in the treatments with essential oil from *S. tupiniquinorum*, although they were different from the positive control.

**Figure 4:**
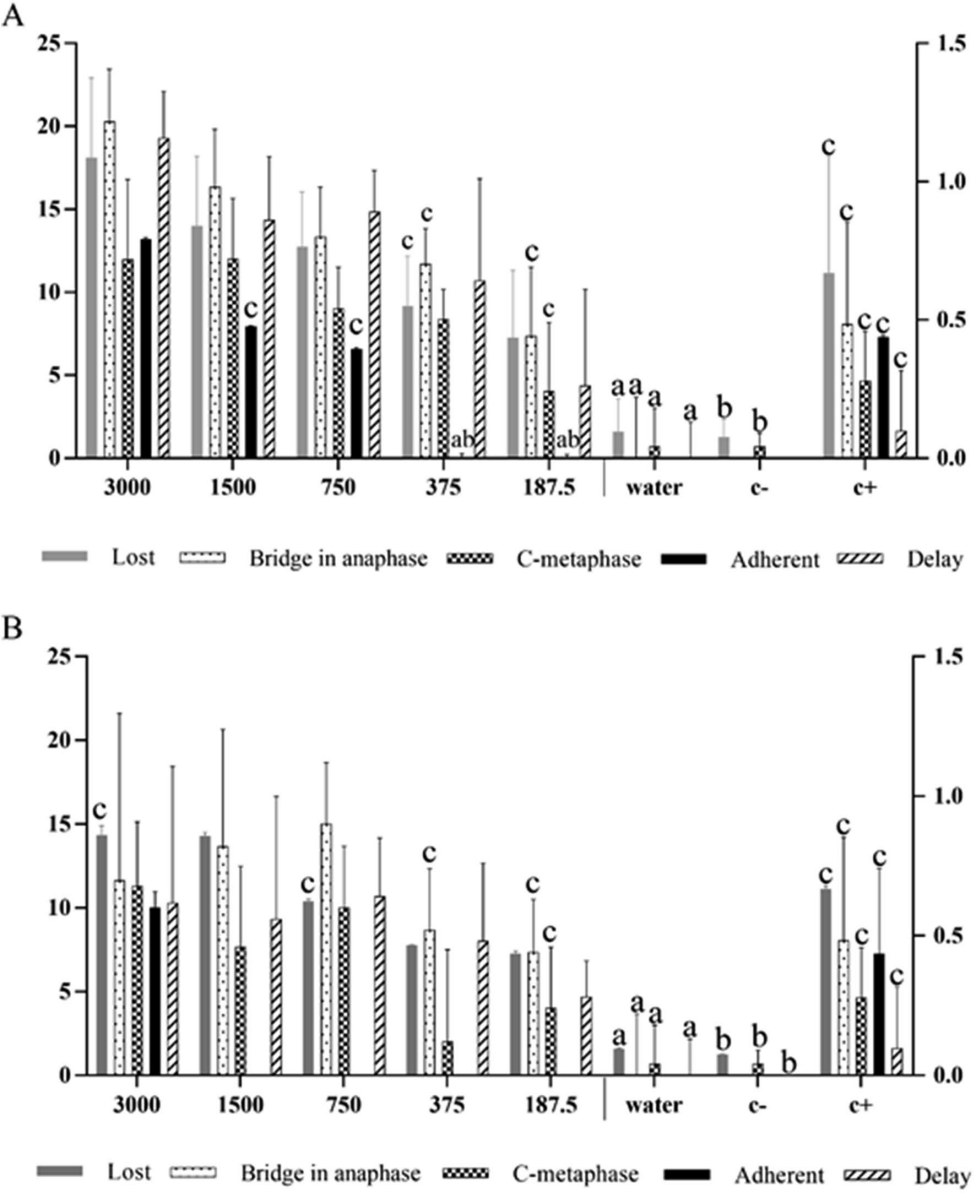
Chromosomal alterations found in *L. Sativa* root meristematic cells treated with the essential oils of **(A)** *S. botocudorum* and **(B)** *S. tupiniquinorum*, water (C −) , solvent (C −) or glyphosate (C +) . The means followed by the letter *a* equalled distilled water (C −); means followed by *b* equalled dichloromethane (DCM) (C −); and means followed by *c* equaled glyphosate (C +) . The five variables shown are lost chromosome, adherent chromosome, anaphase bridge, c-metaphase, and delay in anaphase. The numbers in the figure caption represent the concentrations (ppm) of the essential oils. The left y axis shows the values of lost and adherent chromosomes, and the right y axis refers to the values of anaphase bridge, c-metaphase and delay in anaphase, according to Dunnett’s test (p < 0.05) .

Individual analysis of chromosomal alterations revealed a significant number for the essential oils from both *S. botocudorum* and *S. tupiniquinorum* when compared to the negative controls. Most alterations were similar to or higher than the number found in cells treated with glyphosate (Fig. 4a, b). Lost chromosomes, adherent chromosomes, anaphase bridges, c-metaphases and delay in anaphase were observed.

## Discussion

### Chemical composition of essential oils

Although studies in the genus *Sparattanthelium* are scarce, the chemical composition of its essential oils is similar to other species belonging to the basal angiosperms, mainly from the genus *Hernandia*, family Hernandiaceae. According to Brophy et al.^39^, the species *H. albiflora*, *H. bivalvis* and *H. nymphaeifolia* present bicyclogermacrene, germacrene D and γ-cadinene in their chemical composition. Representing another genus from the same family, *Gyrocarpus americanus* subsp. *Amwlcanu* has germacrene D as major essential oil compound, with 31% of representation^39^. These data indicate that, within the family Hernandiaceae, different species are similar in chemical composition. According to Araujo et al.^40^, the species *Ocotea puberula* (family Lauraceae) presents, among the chemical constituents of its essential oil, bicyclogermacrene, germacrene D, α caryophyllene, β-elemene and γ-cadinene. Other studies performed in the family Piperaceae, another basal angiosperm, have shown that some species have chemical composition similar to the two *Sparattanthelium* species studied here, with divergence only in the major compound^41^.

Studies in species with chemical composition similar to *S. botocudorum* and *S. tupiniquinorum* have shown larvicidal^42^, antibacterial^43,44^, fungicidal^41^ and phytotoxic^45^ activity. The presence of major compounds such as germacrene D and β-elemene also induce biological activities, as observed in *Plantago major* L.^46^, which has bactericidal, antifungal and phytotoxic activity. Transnerolidol, which was only verified in *S*. *botocudorum,* exhibits antileishmanial activity and also hinders the growth of the malaria-causing protozoan *P. falciparum*.

A study of species from the family Lauraceae, presenting bicyclogermacrene in their composition, indicated cytotoxic activity in tumor cells^47^. According to the authors, this compound induces cell apoptosis and may have influenced the observed results, as it may act synergistically with the other identified compounds.

#### Phytotoxicity

The essential oils from *S. botocudorum* and *S. tupiniquinorum* were more efficient in inhibiting variables related to the initial development of lettuce and sorghum seedlings than the commercial herbicide glyphosate. These oils affected the shoot length of both used plant models. In addition, the two model plants, one monocot and one eudicot, were sensitive to even low concentrations of allelochemicals used in the treatments.

Studies report that allelochemicals, when released in sufficient quantities, have effects that can be observed on the germination and development of plant root and shoot systems^48^. Interference in the germination process may affect plant growth and development^49^. Early in the germination process, the glyoxylate cycle is mobilized, supplying enzymes that act on the metabolism of lipids, which are stored in germination tissues, thus favoring plant development^50^. Interference with any of these processes can interrupt germination^49^.

Chemical composition influences the biological activity of essential oils. According to Sampietro^51^, terpenoids are growth inhibitors, whereas monoterpenes and sesquiterpenes are shoot and root growth inhibitors. Monoterpenes can affect the integrity of the cell membrane, causing a change in the fluidity state by acting on membrane phospholipids, increasing the ratio between unsaturated and saturated fatty acids, thus altering the physical arrangement of the membrane^51^. In this study, the essential oils from *S. botocudorum* and *S. tupiniquinorum* inhibited root and shoot growth at some concentrations, which may be due to their chemical composition, mostly composed of germacrene D, bicyclogermacrene and germacrene A.

Alkaloids are another class of compounds present in most species of basal angiosperms. In studies with species of the genus *Ocotea* (family Lauraceae), the chemical composition of the essential oil was found to be rich in alkaloids, which are associated with phytotoxic, larvicidal and antibacterial activities^52^. Isolated alkaloids of *Guatteria* sp. (family Annonaceae) have antitumor, antimalarial, antifungal and mutagenic activities^53^. The chemical composition and biological activity of essential oils from species of basal angiosperms may be related, as their compounds are detoxification products of harmful substances generated by the primary metabolism of plants, acting as allelopathic agents, which can inhibit germination owing to their chelating and/or cytotoxic power^54^.

The germination of lettuce and sorghum seeds showed no significant changes when exposed to the essential oils from *S. botocudorum* and *S. tupiniquinorum*. According to Ferreira and Aquila^14^, the effects of allelochemicals on plants are only a secondary reflection of internal changes. Hence, the impact of allelochemicals on germination and seedling growth are secondary manifestations of effects at the cellular level. In other words, germination occurrence and speed, when analyzed separately, do not always indicate an allelopathic effect, but such an effect can be confirmed by combined analyses at both macroscopic and microscopic levels.

Most of the studies that evaluate the allelopathic effect of plant species analyze variables related to germination and initial growth of test organisms^55–57^. However, many of the visible effects on these variables are signs of changes in DNA that can also be identified in both cytotoxic and cytogenetic assays^58^.

#### Cytotoxicity

Mitotic index and chromosomal and nuclear alterations showed expressive values when compared with the negative controls (water and dichloromethane). These data are closely related to macroscopic and developmental parameters, since the growth of a plant organ is dependent on the number of cells produced during cell division and cell elongation in the process of differentiation and development^25^. In addition, the reduced MI and the frequency of CA and NA affected the growth and development of lettuce and sorghum seedlings in this study.

**Figure 5:**
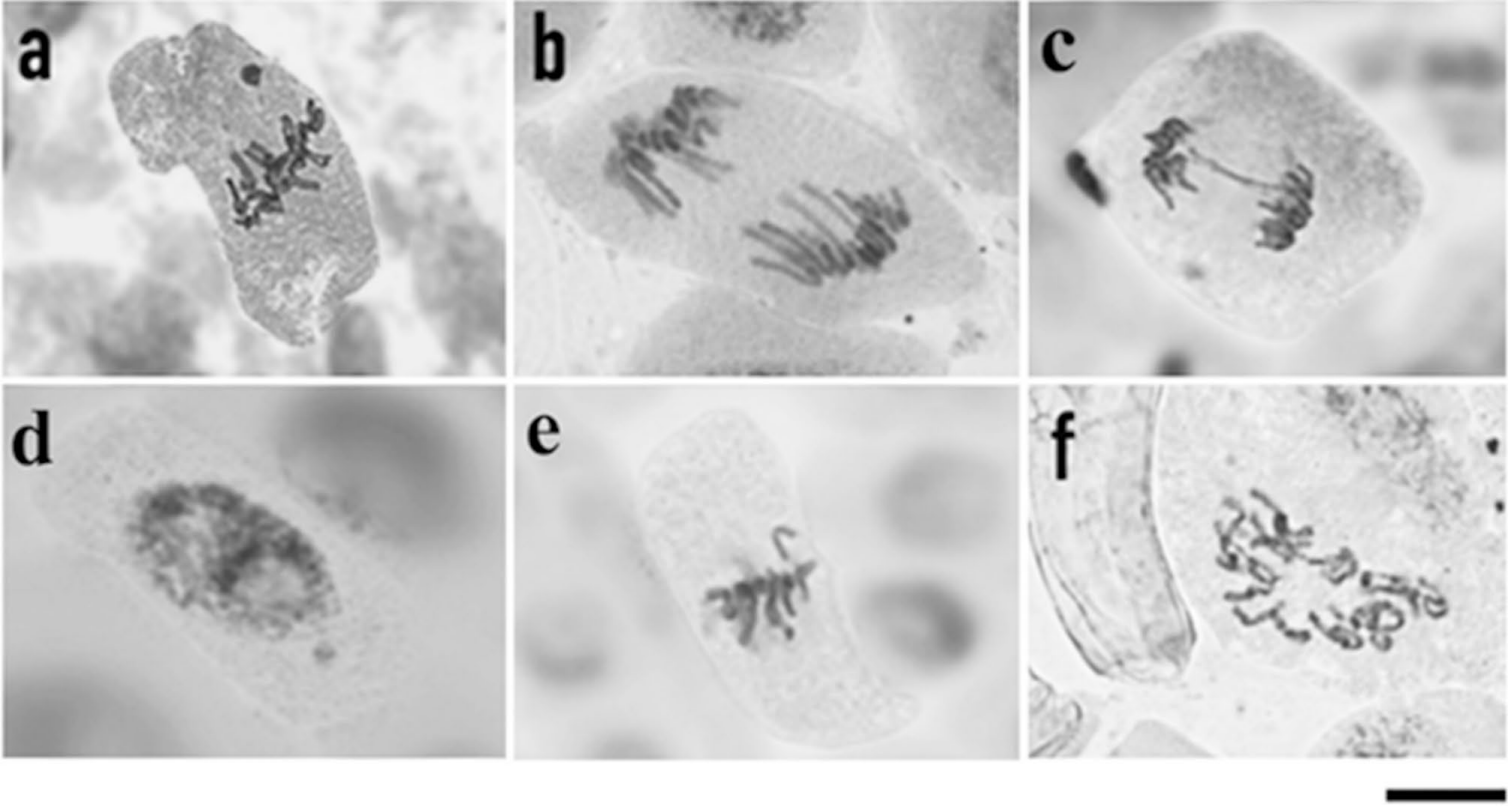
Images of chromosomal and nuclear alterations: **(a)** metaphase micronucleus, **(b)** delay in anaphase, **(c)** anaphase bridge, **(d)** micronucleus in interphase, **(e)** lost chromosome, and **(f)** c-metaphase, observed in meristematic lettuce cells treated with the essential oils of *S. botocudorum* and *S. tupiniquinorum* at 3,000, 1,500, 750, 375 and 187.5 ppm. Bar: 10 μm.

Chromosomal alterations, such as those found here, enable evaluating the mechanisms of action of the essential oil compounds on the cell cycle. These mechanisms may be clastogenic or aneugenic. For instance, the presence of bridges and chromosome fragments demonstrate a clastogenic effect and the action of the molecules directly on the individual's DNA^25,26,59^. Chromosome bridges occur when chromosomes are dragged by their centromere to the cell poles (Fig. 5c); through depolymerization of the alpha and beta tubulin filaments, the breakage of chromosomes forms new chromosome fragments, enabling new fusion and future breaks^27^. Chromosome fragments, also deriving from chromosome breaks, result from interaction of the chemical compound with chromatin/DNA^25,59^. These fragments are acentric, since the breaks occur in telomeric regions, and may lead to the formation of chromosome bridges, thus preventing the attachment of spindle fibers to the chromosome during the cell cycle, since the microtubules bind directly to the centromere^25,27,59^.

Alterations such as lost chromosomes, micronuclei, delay in anaphase and c-metaphases characterize aneugenic action (i.e. inhibition of mitotic spindle fibers) of the chemical substances. Both lost chromosomes and fragments can result in micronuclei. Oliveira^60^ reported that the frequency of micronuclei observed in root meristem cells of *Allium cepa* indicates the action of aneugenic substances. These alterations are closely related to the morphological responses of plants and their growth, development and photosynthesis. Aneugenic compounds act directly on the DNA, inhibiting the repair mechanism of the cell. The delay in anaphase is also related to depolymerization of the microtubules, resulting in uneven dragging of the chromosomes. In addition, it causes the cell to have a deformed nuclear membrane, aiming to envelop all the genetic material to it and then the deformation is undone^59^. In c-metaphases, the microtubules are completely inactivated, preventing the spindle formation^61,62^, i.e., the mitotic spindle becomes completely inactivated, disorganizing the formation of the equatorial plate, thus impeding or delaying the division of the centromere^61,62^. The continued presence of c-metaphases can lead to duplication in the number of chromosomes of these cells.

Another alteration found was chromosome adhesion, characterized by irreversible attachment of chromosomes, which likely leads to cell death. It is an alteration indicative of several mechanisms of action simultaneously, and may be aneugenic, clastogenic and epigenetic^27,61,63^.

## Conclusion

The essential oils from *S. botocudorum* and *S. tupiniquinorum* showed phytotoxic effects on both monocot and eudicot specimens. In addition, they demonstrated aneugenic and clastogenic mechanisms of action, promoting DNA and mitotic spindle damage. Therefore, the essential oils of these species are promising among the choices for new substances with potential biological activities.
